# Dopamine Inhibits Mitochondrial Motility in Hippocampal Neurons

**DOI:** 10.1371/journal.pone.0002804

**Published:** 2008-07-30

**Authors:** Sigeng Chen, Geoffrey C. Owens, David B. Edelman

**Affiliations:** The Neurosciences Institute, San Diego, California, United States of America; National Institutes of Health, United States of America

## Abstract

**Background:**

The trafficking of mitochondria within neurons is a highly regulated process. In an earlier study, we found that serotonin (5-HT), acting through the 5-HT1A receptor subtype, promotes axonal transport of mitochondria in cultured hippocampal neurons by increasing Akt activity, and consequently decreasing glycogen synthase kinase (GSK3β) activity. This finding suggests a critical role for neuromodulators in the regulation of mitochondrial trafficking in neurons. In the present study, we investigate the effects of a second important neuromodulator, dopamine, on mitochondrial transport in hippocampal neurons.

**Methodology/Principal Findings:**

Here, we show that dopamine, like 5-HT, regulates mitochondrial motility in cultured hippocampal neurons through the Akt-GSK3β signaling cascade. But, in contrast to the stimulatory effect of 5-HT, administration of exogenous dopamine or bromocriptine, a dopamine 2 receptor (D2R) agonist, caused an inhibition of mitochondrial movement. Moreover, pretreatment with bromocriptine blocked the stimulatory effect of 5-HT on mitochondrial movement. Conversely, in cells pretreated with 5-HT, no further increases in movement were observed after administration of haloperidol, a D2R antagonist. In contrast to the effect of the D2R agonist, addition of SKF38393, a dopamine 1 receptor (D1R) agonist, promoted mitochondrial transport, indicating that the inhibitory effect of dopamine was actually the net summation of opposing influences of the two receptor subtypes. The most pronounced effect of dopamine signals was on mitochondria that were already moving directionally. Western blot analysis revealed that treatment with either a D2R agonist or a D1R antagonist decreased Akt activity, and conversely, treatment with either a D2R antagonist or a D1R agonist increased Akt activity.

**Conclusions/Significance:**

Our observations strongly suggest a role for both dopamine and 5-HT in regulating mitochondrial movement, and indicate that the integrated effects of these two neuromodulators may be important in determining the distribution of energy sources in neurons.

## Introduction

It has been shown that axonal transport of mitochondria ensures proper neuronal function [1]. Aberrant mitochondrial trafficking has been linked to a number of neurodegenerative disorders, including Alzheimer's disease, Parkinson's disease, Huntington's disease, and Lou Gehrig's disease [2]–[4]. Although several signals have been found to modulate mitochondrial trafficking in neurons [5]–[8], the precise roles of these signals have not been fully characterized.

Previously, we identified serotonin (5-HT) as an extracellular signal that can promote axonal mitochondrial movement in cultured hippocampal neurons, suggesting a possible relationship between neuronal activity and mitochondrial movement [9]. In the same study, we demonstrated the importance of the Akt-GSK3β signaling cascade in the control of mitochondrial trafficking in response to 5-HT signaling. Direct inhibition of Akt suppressed mitochondrial trafficking, whereas inhibition of GSK3β, which is directly phosphorylated, and thus inhibited, by Akt, enhanced trafficking [9]. These results indicate a general role for this pathway in the control of mitochondrial movement, and suggest that other signals converging on the Akt-GSK3β pathway may also affect the trafficking of mitochondria. Interestingly, dopamine has recently been shown to act through the Akt-GSK3β pathway in striatal neurons [10]–[12].

Dopamine is an important neurotransmitter that is involved in many aspects of neural function, including motor activity, emotion, reward, sleep, and learning [13]–[15]. Although it has been suggested that the effects of dopamine in disorders such as Parkinson's disease and schizophrenia are linked to impaired mitochondrial function [16], [17], an influence of dopamine on mitochondrial movement has not been reported.

With the foregoing considerations in mind, we decided to investigate the effect of dopamine on mitochondrial trafficking. Based on the analysis of data from time-lapse imaging of cultured hippocampal neurons, we report here that dopamine has a net inhibitory effect on mitochondrial movement. Specifically, whereas activation of the D2 receptor inhibited the movement of mitochondria, activation of the D1 receptor promoted the movement of mitochondria. Consistent with their effects on mitochondrial motility, dopamine agonists and antagonists also showed opposing effects on the Akt-GSK3β signaling cascade, the same pathway that is activated by 5-HT in modulating mitochondrial motility in hippocampal neurons [9]. When we stimulated mitochondrial movement with 5-HT, then added a D2R agonist, movement was strongly reduced. However, treatment with a D1R agonist did not cause an increase in trafficking above that induced by 5-HT. These observations point to a possible physiological role for both dopamine and 5-HT in regulating mitochondrial movement in hippocampal neurons. Previously, it has been suggested that dopamine and 5-HT signals can interact coordinately to regulate neuronal activity in the striatum [11], [18]; however, the coordinate regulation of mitochondrial motility by these two neurotransmitters in hippocampal neurons is a novel finding.

## Results

### Dopamine inhibits mitochondrial motility in hippocampal neurons

**Figure 1:**
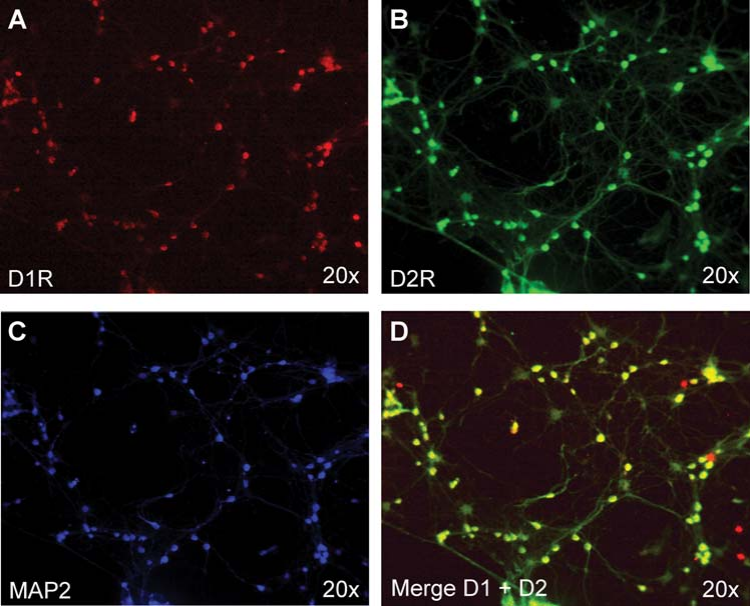
The dopamine receptor subtypes, D1R and D2R, are both expressed in cultured hippocampal neurons (A–D). D1 receptors are shown in red (A); D2 receptors are shown in green (B); MAP2 expression (soma and dendrites) is shown in blue (C); the merged image (D) indicates colocalization (yellow).

**Figure 2:**
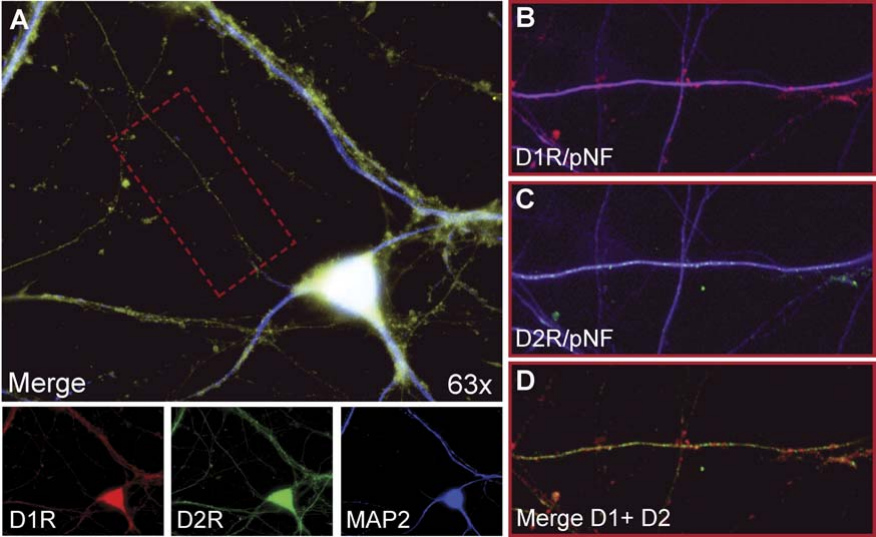
Both dopamine receptor subtypes are expressed in the same hippocampal neurons (A–D). A. A typical pyramidal neuron that was used in mitochondrial motility studies shows expression of both D1 and D2 receptors (A, top panel). D1 receptors (D1R) are shown in red (bottom, left panel); D2 receptors (D2R) are shown in green (bottom, middle panel); MAP2 expression (soma and dendrites) is shown in blue (bottom, right panel). The merged image (A) indicates colocalization (yellow). B–D. Detail of expression of D1R (B) and D2R (C) in axons (enlargement of rectangular region indicated by red dashed border in A); merged image of D1R and D2R labeling is shown in D (labeling of axon has been removed for clarity). Axons in B and C are immunolabeled with phospho-neurofilament (pNF) antibody (purple).

As described in our earlier study of 5-HT and mitochondrial transport [9], we employed fully differentiated and spontaneously active hippocampal neurons as a model culture system. Consistent with the finding that the dopamine receptor subtypes, D1 and D2, colocalize in neostriatal neurons [19], we found a similar pattern of expression in nearly all of the cultured hippocampal neurons that we imaged (Fig. 1A–D and Fig. 2A), including evidence of punctate immunoreactivity associated with axons (Fig. 2B–D). Cultures were infected with the Flx1.8/MitoEYFP lentivirus [9] and used in live imaging experiments. Images were collected for 2 hours prior to the addition of dopamine (30 nM), and then for 2 hours after exposure to dopamine (representative time-lapse movies are available in Supporting Information). Following the same criteria used previously, patterns of mitochondrial motility during long-term observation were categorized into three distinct groups: a stationary population, an oscillatory population, and a directionally moving population [9]. A mitochondrion was classified as stationary if it moved less than 0.2 µm (equivalent to 2 pixels; 1 pixel = 0.10235 um under 63× magnification) over the period of observation. A mitochondrion was considered to be oscillatory if it moved bidirectionally at least 0.2 µm—but not more than 2.5 µm—around a fixed point during the period of observation. A mitochondrion was considered to move directionally, either anterogradely or retrogradely, if it was observed to travel a distance greater than 2.5 µm within the axon. The same classification scheme was also applied in the analysis of dendritic mitochondria (data not shown).

**Figure 3:**
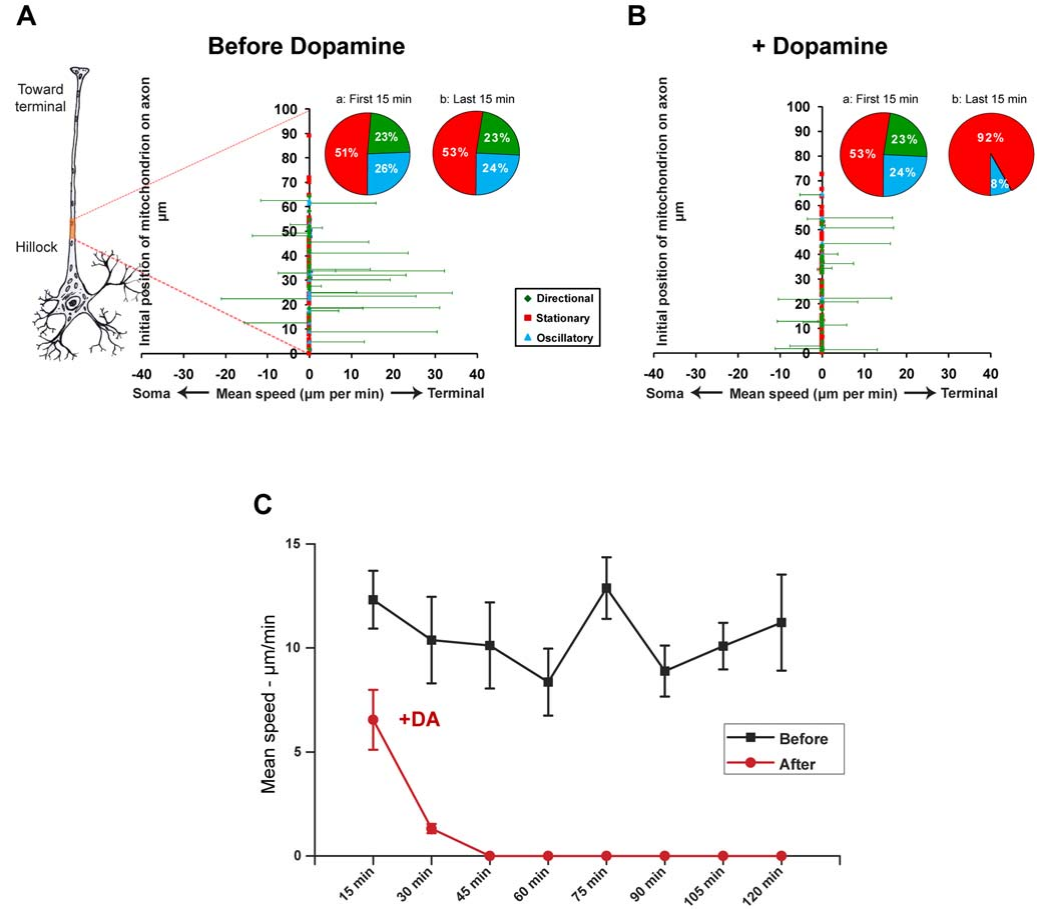
Dopamine inhibits axonal transport of mitochondria in hippocampal neurons (A–C). A, B. Mean speeds (µm/min) of individual mitochondria before (A) and after (B) treatment with dopamine (30 nM). Pie chart insets show the percentage of stationary (red), oscillatory (blue), and directionally moving (green) mitochondria in all pooled experiments in the initial (a) and final (b) 15 minutes of imaging. C. Changes in mean speeds of all directionally moving mitochondria over time following treatment with dopamine. “(−)” indicates retrograde movement; “(+)” indicates anterograde movement. The red dotted lines projecting from the highlighted region of the schematic axon to the Y-axis of each graph in A and B indicate the approximate location (∼100–150 µm from the soma) and extent (∼50–80 µm) of the axon segment that was imaged.

Following a method described previously [9], the mean speed of each individual mitochondrion in the axonal segment under observation was calculated from the cumulative distance traveled over the entire period of observation. The initial relative position of each mitochondrion along the axonal segment is shown on the y-axis in Fig. 3A and B. The average numbers of stationary, oscillatory, and directionally moving mitochondria were obtained from time-lapse images taken in the first and last 15 minutes of each experiment (pie chart insets, Fig. 3A, B). Prior to treatment with dopamine (30 nM), most axonal mitochondria (∼77%) were either stationary or moved in an oscillatory manner (Fig. 3A, pie chart insets a and b; Movie S1). Following the administration of dopamine, the directionally moving mitochondria slowed down dramatically, and at the end of two hours, all directional movement was inhibited (compare Fig. 3A, B, and corresponding pie chart insets; Movies S1 and S2). The oscillatory population was also significantly diminished. To determine the time of onset of the effect of dopamine on mitochondrial motility, the mean speeds of all directionally moving mitochondria were binned every 15 minutes two hours before and two hours after treatment. As shown in Fig. 3C, there was already a dramatic decrease in the rate of mitochondrial movement 15 minutes after the administration of dopamine. Within 45 minutes following treatment, virtually all mitochondria had essentially stopped moving. Each data set was obtained from five paired time-lapse imaging experiments (five separately prepared cultures, each imaged for 2 h before and 2 h after dopamine treatment; a total of 236 mitochondria tracked).

Given the observed inhibitory effect of dopamine on mitochondrial motility, we were concerned that the mitochondria in our cultured neurons had been directly impaired by exposure to exogenous monoamine. In other studies of mitochondrial motility, inhibitory effects on mitochondrial movement have generally been consistent with mitochondrial impairment, indicated by a loss of membrane potential and drastic changes in mitochondrial morphology [7], [8], [20], [21]. However, we did not observe any obvious signs of toxicity in neurons treated with the amount of dopamine that we administered; nor did we see any change in mitochondrial membrane potential as visualized with the vital dye JC-1 (Movie S3). As expected, when carbonylcyanide p-trifluoromethoxyphenylhydrazone (FCCP), an uncoupler, was added to a parallel culture as a positive control, JC-1 fluorescence was extinguished over time, indicating a loss of membrane potential (Movie S4).

### A D2R agonist inhibits mitochondrial transport, whereas a D2R antagonist promotes mitochondrial transport

**Figure 4 pone-0002804-g004:**
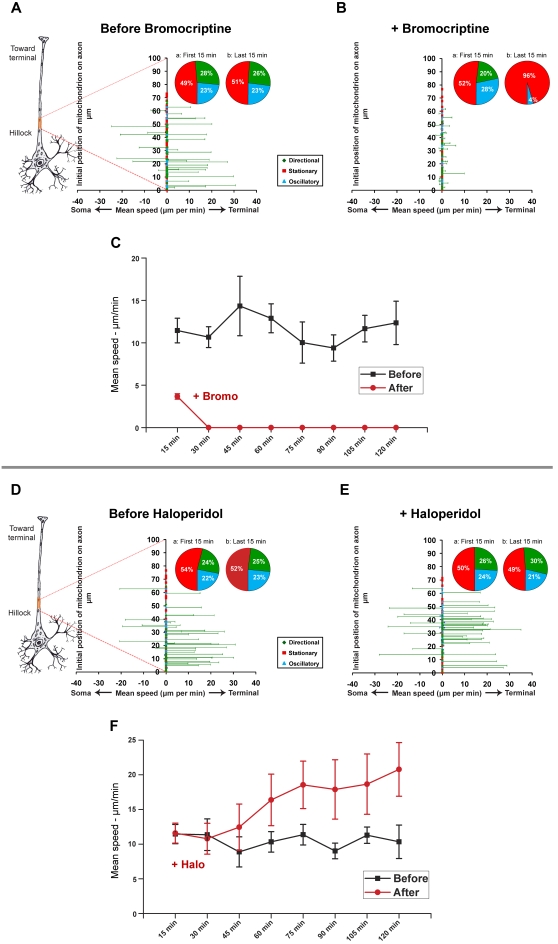
Bromocriptine, a D2R agonist, inhibits axonal transport of mitochondria in hippocampal neurons, whereas Haloperidol, a D2R antagonist, promotes mitochondrial transport (A–F). A–B and D–E. Mean speeds (µm/min) of individual mitochondria before and after treatment with Bromocriptine (5 µM; A,B) or Haloperidol (10 µM; D,E). Pie chart insets show the percentage of stationary (red), oscillatory (blue), and directionally moving (green) mitochondria in all pooled experiments in the initial (a) and final (b) 15 minutes of imaging. C,F. Changes in mean speeds of all directionally moving mitochondria over time following treatment with D2R agonists and antagonists. “(−)” indicates retrograde movement; “(+)” indicates anterograde movement. The red dotted lines projecting from the highlighted region of the schematic axon to the Y-axis of each graph indicate the approximate location (∼100–150 µm from the soma) and extent (∼50–80 µm) of the axon segment that was imaged.

Administration of the dopamine 2 receptor (D2R) agonist, bromocriptine (5 µM), in the absence of added dopamine markedly inhibited both directional and oscillatory movement of mitochondria (compare Fig. 4A and B, pie chart insets a and b; Movies S5 and S6). Based on an analysis of time-lapse image sequences from five separate paired experiments (five separately prepared cultures, each imaged for 2 h before and 2 h after treatment with bromocriptine; a total of 227 mitochondria tracked), it appeared that activation of the D2 receptor induced mitochondria that had previously been moving directionally to stop moving entirely. As shown in Fig. 4C, bromocriptine showed a dramatic dampening effect on mitochondrial motility within 15 minutes following treatment (Fig. 4C).

To further investigate the role of the D2 receptor in regulating mitochondrial movement, we examined the effects of a D2R antagonist on cultured hippocampal neurons. In contrast to the effect of bromocriptine, administration of the D2R antagonist, haloperidol (10 µM), in the absence of added dopamine significantly stimulated mitochondrial movement; the majority of this movement was anterograde (e.g., toward the axonal terminal) (Fig. 4D, E; Movies S7 and S8). Although an analysis of images from six paired time-lapse imaging experiments (six separately prepared cultures, each imaged for 2 h before and 2 h after haloperidol treatment; a total of 288 mitochondria tracked) did not show much increase in the size of the directionally moving population (Fig. 4D, E; pie chart insets a and b), starting at 30 minutes, there was a marked increase in the average speed of all directionally moving mitochondria (Fig. 4F) that reached 1.8-fold of the control population after 2 hours (n = 6, paired *t*-test; p<0.02). This result suggests that haloperidol blocked the effects of any endogenous dopamine in the cultures. In earlier studies, dopamine uptake by cultured rat astrocytes was demonstrated [22], [23], suggesting that astrocytes might be a possible source of endogenous dopamine in our culture system, in which we use glia-conditioned media.

### A D1R agonist promotes mitochondrial transport, whereas a D1R antagonist inhibits mitochondrial movement

**Figure 5:**
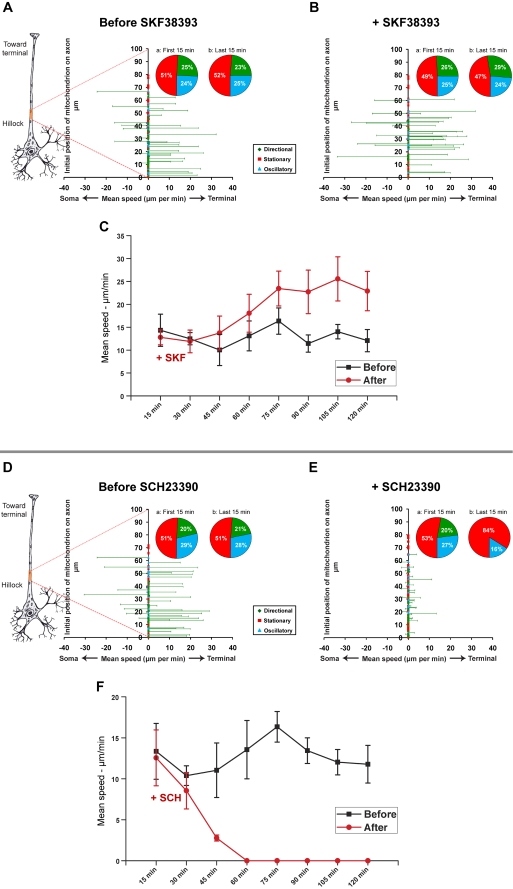
SKF38393, a D1R agonist, promotes axonal transport of mitochondria in hippocampal neurons, whereas SCH23390, a D1R antagonist inhibits mitochondrial transport (A–F). A–B and D–E. Mean speeds (µm/min) of individual mitochondria before and after treatment with SKF38393 (33 µM; A,B) or SCH23390 (55 µM; D,E). Pie chart insets show the percentage of stationary (red), oscillatory (blue), and directionally moving (green) mitochondria in all pooled experiments in the initial (a) and final (b) 15 minutes of imaging. C,F. Changes in mean speeds of all directionally mitochondria over time following treatment with D1R agonists and antagonists. “(−)” indicates retrograde movement; “(+)” indicates anterograde movement. The red dotted lines projecting from the highlighted region of the schematic axon to the Y-axis of each graph in A and B and E and F indicate the approximate location (∼100–150 µm from the soma) and extent (∼50–80 µm) of the axon segment that was imaged.

D1 receptors are the second major class of dopamine receptors found in hippocampal neurons (Figs. 1, 2). Since signals from D2R and D1R may have opposing effects [19], [24], we decided to investigate the possible effect of a D1R-specifc agonist on mitochondrial movement. Administration of the D1R agonist, SKF38393 (33 µM), markedly stimulated mitochondrial movement, primarily anterogradely, toward the axon terminal (Fig. 5A, B; Movies S9 and S10). Analysis of six paired time-lapse imaging experiments (six separately prepared cultures, each imaged for 2 h before and 2 h after treatment with SKF38393; a total of 252 mitochondria tracked) showed a slight increase in the size of the directionally moving population (Fig. 5B, pie chart insets a and b). There was also a slight increase in the mean speed of directionally moving mitochondria approximately one hour following the treatment. After 2 hours, the average speed of all directionally moving mitochondria had increased by 65% compared to the 2 hours prior to drug treatment (Fig. 5C; n = 6, paired *t*-test; p<0.02).

In contrast, an analysis of five paired time-lapse imaging experiments (five separately prepared cultures, each imaged for 2 h before and 2 h after treatment with SCH23390; a total of 287 mitochondria tracked) showed that administration of the D1R antagonist, SCH23390 (55 µM), inhibited virtually all directional movement of mitochondria within one hour, and dramatically decreased oscillatory movement (Fig. 5D, E; pie chart insets a and b; 5F; Movies S11 and S12).

### Mitochondrial transport is reversibly modulated by sequential activation of D2R and D1R receptors

**Figure 6:**
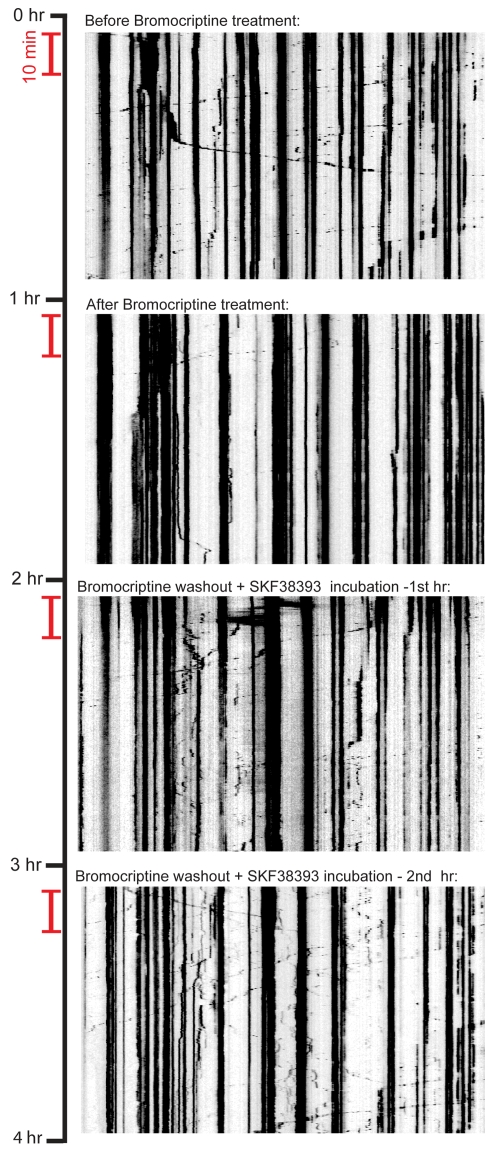
Modulation of mitochondrial movement by D2R and D1R agonists is reversible. Kymographs of mitochondrial motility shown in supplementary movies (Movies S13-S16); vertical scale bar = 10 minutes. In the experiment, a segment of axon was imaged continuously for four hours (with short breaks to allow for exchange of media and administration of drugs) at 10-second intervals. Hour 1: before treatment, basal level of mitochondrial movement. Hour 2: treatment with bromocriptine. Hour 3: washout of bromocriptine, and addition of SKF38393. Hour 4: one hour incubation with SKF38393.

As shown in Figures 1 and 2, we found that both D2R and D1R are expressed in the same hippocampal neurons. Since dopamine, which stimulates both D1R and D2R, blocks mitochondrial movement, a D2R-dependent ‘stop’ signal must predominate over a D1R-mediated ‘go’ signal. We wished to determine whether the effect of D2R activation was reversible, and whether inhibited mitochondria could be re-induced to move by subsequent activation of D1R within the same neuron. When we sequentially added bromocriptine, and then SKF38393, after washout of the D2R agonist, we observed that mitochondrial movement was inhibited and then subsequently enhanced following exposure to the D1R agonist. Addition of SKF38393 failed to restore mitochondrial movement without washing out the bromocriptine, the D2R agonist (data not shown). In this experiment, we employed a red fluorescent protein, which allowed us to acquire images continuously every 10 seconds over a period of 4 hours without any observable phototoxicity (Movies S13-S16). To illustrate this result, we present the data as kymographs (Fig. 6). This experiment demonstrates unambiguously that mitochondrial movement can be reversibly modulated by differential activation of the two dopamine receptor subtypes.

### The Akt-GSK3β signaling pathway is involved in dopamine-mediated regulation of mitochondrial movement

**Figure 7:**
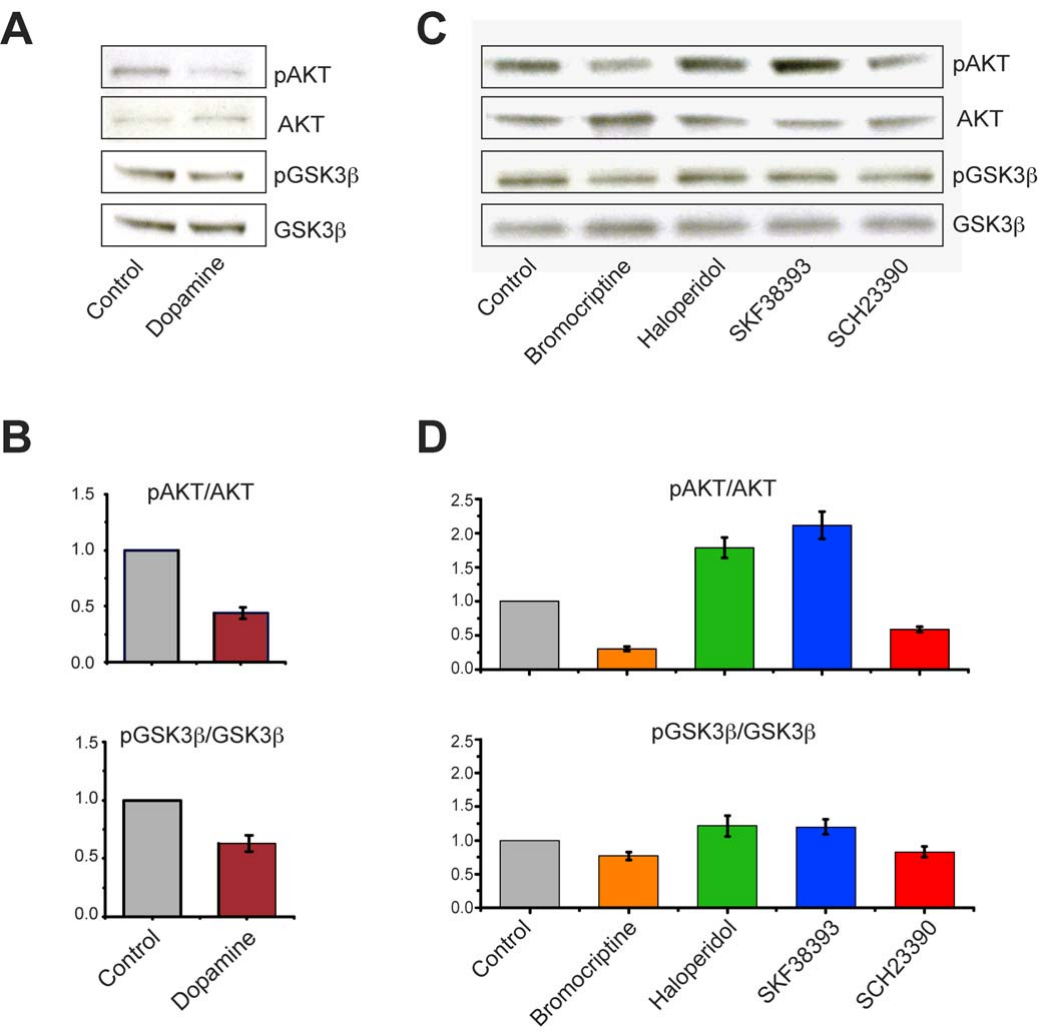
Dopamine signals are coupled to the activation of Akt and inactivation of GSK3β in hippocampal neurons (A–D). A. Western blot analysis shows that treatment with dopamine (30 nM) for 60 minutes leads to the inactivation of Akt (i.e., decreased serine-473 phosphorylation) and the activation of GSK3β (i.e., decreased serine-9 phosphorylation). B. Quantification of Western blots (n>3) showing the effect of dopamine alone on the phosphorylation of Akt (top) and GSK3β (bottom). C. Treatment with a D2R agonist (Bromocriptine, 5 µM) or a D1R antagonist (SCH23390, 55 µM) for 60 minutes leads to the inactivation of Akt and the activation of GSK3β, whereas treatment with a D2R antagonist (Haloperidol, 10 µM) or a D1R agonist (SKF23383, 33 µM) for 60 minutes leads to the activation of Akt and inactivation of GSK3β. D. Quantification of Western blots (n>3) showing the effect of dopamine signals on the phosphorylation of Akt (top) and GSK3β (bottom). Whole cell extracts from hippocampal neuronal cultures (DIV 17) were used in Western blots. In B and D, intensity values were normalized to total Akt or total GSK3β values.

Previously, using inhibitors of Akt or GSK3β and phospho-specific antibodies in a Western blot analysis, we showed that the Akt-GSK3β signaling pathway is explicitly involved in the modulation of mitochondrial movement in response to stimulation by 5-HT [9]. We employed the same method here to determine if the Akt-GSK3β signaling cascade might also modulate the effects of dopamine on mitochondrial movement. Western blot analysis showed that the Akt-GSK3β signaling pathway is strongly implicated in dopamine signaling in hippocampal neurons treated with different dopamine agonists and antagonists. When neurons were treated with dopamine alone (30 nM), levels of activated Akt (pAkt) decreased by 50% (Fig. 7A, B). In agreement with our imaging data (Fig. 3), the effect of dopamine administration on the phosphorylation of Akt was evident after 15 minutes (Fig. S1). Treatment with the D2R agonist, bromocriptine (5 µM; Fig. 7C, lane 2), or the D1R antagonist, SCH23390 (55 µM; Fig. 7C, lane 5) also decreased levels of pAkt by more than 75% and 50%, respectively (Fig. 7D, top panel, columns 2 and 5). As well, after treatment with bromocriptine or SCH23390, levels of inactivated GSK3β (pGSK3β) were decreased by 25% (Fig. 7D, bottom panel, columns 2 and 5). Conversely, treatment with the D2R antagonist, haloperidol (10 µM; Fig. 6C, lane 3), or the D1R agonist, SKF38393 (33 µM; Fig. 7C, lane 4), increased levels of both pAkt and pGSK3β, by 75% and 30%, respectively (Fig. 7D, bottom panel, columns 3 and 4).

**Figure 8:**
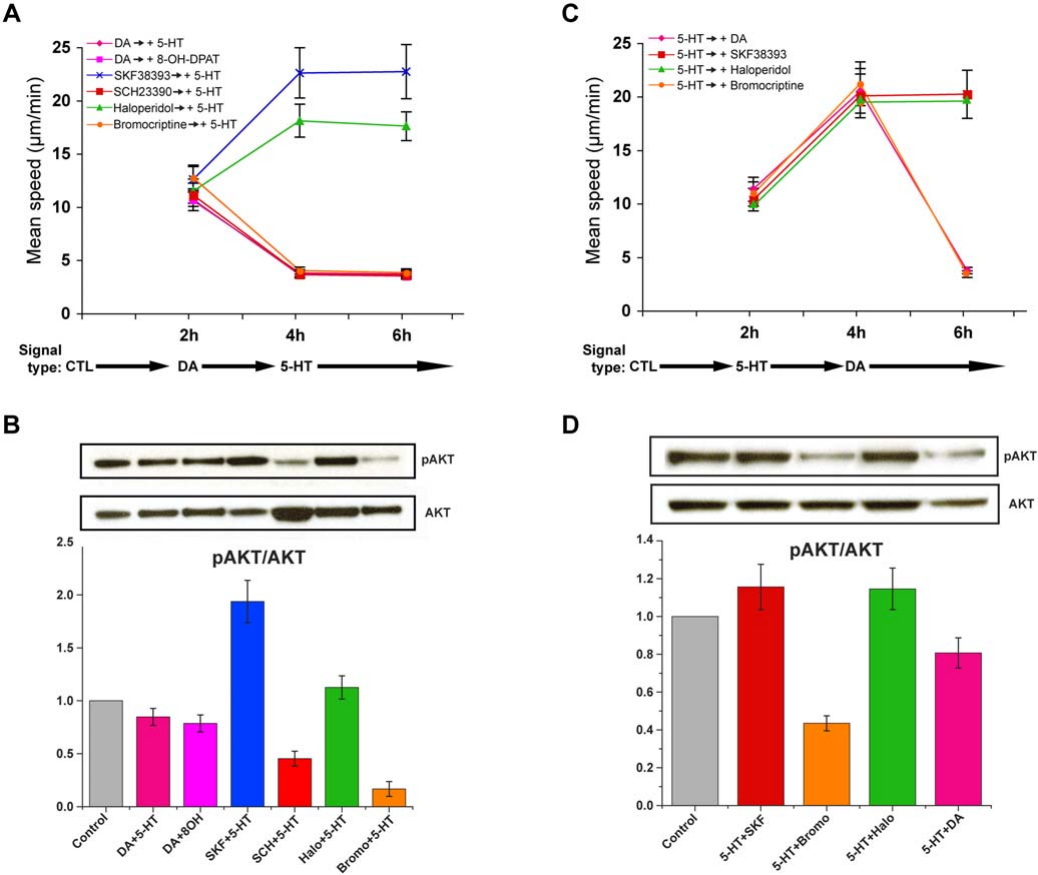
Treatment with IBMX has different effects on hippocampal neurons and striatal neurons (A–C). A. Inhibition of mitochondrial movement by IBMX. Mean speed (µm/min) of all directionally moving mitochondria from pooled experiments before and after treatment with IBMX (100 µM; n = 5, paired *t*-test; p<0.02). B. Western blot analysis shows that administration of IBMX (100 µM) reduces Akt activity in hippocampal neurons, which is represented by decreased levels of phosphorylated serine-473 of Akt, relative to total Akt, over time. C. Western blot analysis shows that administration of IBMX (100 µM) increases Akt activity in striatal neurons, which is represented by decreased levels of phosphorylated serine-473 of Akt, relative to total Akt, over time.

Given the importance of the cAMP pathway in dopamine signaling [24], [25], we decided to elevate cAMP levels directly in hippocampal neurons by treating cultures with 3-isobutyl-1-methylxanthine (IBMX, 100 µM). In the striatum, D1R signaling is coupled to an increase in adenylyl cyclase activity [24], [25]; we therefore anticipated that IBMX treatment might cause an increase in mitochondrial movement. However, as shown in Figure 8A, the mean speed of mitochondrial movement (averaged over the entire imaging session) was dramatically reduced (Movies S17 and S18). Moreover, within 15 minutes, treatment with IBMX led to a decrease in Akt activity that was sustained over at least 60 minutes (Fig. 8B). These results are consistent with the idea that dopamine receptors in hippocampal neurons are coupled to a different downstream signaling pathway than the same receptors in striatal neurons. In support of this interpretation, when we treated striatal neurons with IBMX, we observed an increase in Akt activation, as previously reported [26] (Fig. 8C).

### 5-HT and dopamine exert opposing effects in the regulation of mitochondrial movement

**Figure 9:**
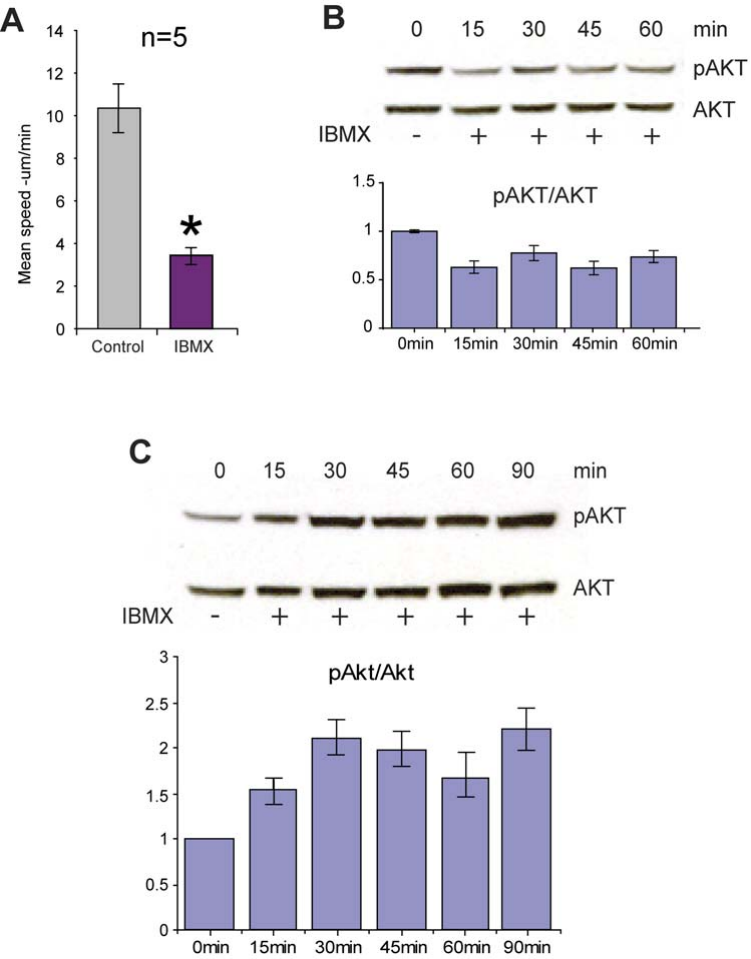
Dopamine and 5-HT exert opposing effects on mitochondrial movement and Akt activity (A–D). Mean speeds of directionally moving mitochondrial populations are shown at two hour intervals in A and C. Akt activity levels in cultures after treatment with DA and 5-HT are shown in B and D. Mitochondrial movement was measured following serial administration of dopamine or its receptor agonists, antagonists, and 5-HT. In these experiments, a 6 hour period of observation was divided into three two-hour intervals, each allocated for a different treatment, i.e., control (CTL), dopamine (DA), or 5-HT. A. Prior to treatment with 5-HT, hippocampal neurons were pretreated with dopamine receptor-specific agonists or antagonists, including DA, SKF38393, SCH2390, haloperidol, and bromocriptine. B. Western blot analysis shows that dopamine counteracts the effect of 5-HT on Akt activity in the presence of 5-HT. C. Prior to treatment with dopamine signals, hippocampal neurons were pretreated with 5-HT. Each mean speed value in the graph represents three repeat experiments, i.e., separately prepared cultures (n = 3, paired *t*-test; p<0.02; 103–130 mitochondria were tracked in calculating the average speeds of each set of mitochondria in a given treatment interval). D. Western blot analysis shows that 5-HT failed to counteract the effect of dopamine on Akt activity in the presence of dopamine. All other conditions are the same as previously described in Figs. 3–5.

We have shown previously that exogenous 5-HT promotes mitochondrial trafficking in cultured hippocampal neurons [9]. In the present study, we found that dopamine exerts a net inhibitory effect on mitochondrial movement in the same model system (Fig. 3). It is therefore possible that the two neurotransmitters have opposing effects on mitochondrial movement, given that the Akt-GSK3β signaling pathway is affected by both signals. To explore this possibility, we performed experiments in which 5-HT and dopamine receptor agonists and antagonists were added sequentially in different combinations to the same culture and their cumulative effects on mitochondrial movements were observed over time using time-lapse fluorescence microscopy (Figs. 9A, C) and by Western blot analysis to detect activated Akt levels under the same conditions (Figs. 9B, D). The results of these experiments showed that inhibition of mitochondrial movement by dopamine, a D2R agonist, or a D1R antagonist was not reversed by subsequent addition of 5-HT (Fig. 9A). Moreover, the increased speed of moving mitochondria induced by treatment with either a D2R antagonist or a D1R agonist was not further enhanced by subsequent treatment with 5-HT (Fig. 9A). Western blot analysis of cultures treated with the same drug regimen showed that Akt phosphorylation remained below control levels in the presence of dopamine, bromocriptine, or SCH23390 even after addition of 5-HT. In an earlier study, we showed that the addition of 5-HT increases Akt phosphorylation [9]. In the present study, Akt phosphorylation remained elevated in the presence of either SKF38393 or haloperidol and 5-HT (Fig. 9B). In contrast to these observations, when hippocampal neurons were pretreated with 5-HT, the subsequent addition of a dopamine D2R agonist led to a marked inhibitory effect on mitochondrial movement (Fig. 9C). However, neither the D2R antagonist nor the D1R agonist stimulated mitochondrial movement beyond levels observed after administration of 5-HT alone (Fig. 9C). As shown in Figure 9D, the addition of dopamine or bromocriptine caused a decrease in Akt phosphorylation, whereas Akt phosphorylation again remained elevated in the presence of either SKF38393 or haloperidol and serotonin. To confirm that the effect of dopamine or bromocriptine was reversible, these drugs were washed out before adding 5-HT or the D1R agonist, SKF38393 (Fig. S2).

## Discussion

### Dopamine has a net inhibitory effect on mitochondrial movement in hippocampal neurons

Dopamine is an important neurotransmitter in the regulation of many aspects of neural function. Changes in levels of dopamine, as well as in the dynamic activity of mitochondria, have been implicated in the onset of symptoms in both Parkinson's disease and schizophrenia [16], [17]. Hitherto, no direct observations have been reported of the effect of dopamine on mitochondrial trafficking. In the present study, we found that dopamine dramatically inhibited mitochondrial movement in cultured hippocampal neurons. Although we observed a similar net inhibitory effect of dopamine on mitochondrial movement in dendrites, the high density of mitochondria at the proximal ends of these processes, as well as possible fission and fusion events at their distal ends, prevented us from performing precise quantification.

A number of studies have reported that dopamine is toxic to neurons *in vitro* [27], [28]. However, neurotoxicity likely follows from exposure to relatively high amounts of dopamine [28], well in excess of physiologically relevant dosages. In our studies, we used a concentration (30 nM) which is considered to be within the physiological range [29]. Moreover, the length of exposure in our experiments was limited to between two and four hours. We observed no change in the normal membrane potential of mitochondria, as evidenced by fluorescence of the vital dye, JC1 (Movies S3 and S4); hence, the inhibitory effect of dopamine on mitochondrial movement that we observed is unlikely to be attributable to an obvious impairment of mitochondria per se. Moreover, administration of 5-HT, SCH23390, or SKF38393 (as well as the other drugs used in the present study) did not induce changes in mitochondrial membrane potential in hippocampal neurons (unpublished data). This finding is consistent with a published study that employed SH-SY5Y cells [28].

We found that dopamine has a net inhibitory effect on mitochondrial movement in hippocampal neurons (Fig. 3A–C). Subsequent experiments with different receptor-specific agonists and antagonists revealed that this observed inhibition likely reflects the net outcome of the opposing effects of different dopamine receptor subtypes within the same cells. Specifically, a D2R agonist inhibited mitochondrial movement (Fig. 4A–C), whereas a D1R agonist promoted mitochondrial movement (Fig. 5A–C). The extent of overlap between D1 and D2 receptors in another brain region, the striatum, is still not completely resolved [30], although recent work has shown that there is a substantial overlap of expression in medium spiny neurons [31]. The results of our immunocytochemistry experiments, in which we used receptor-specific antibodies that had been previously employed by others [19], indicate that, like neostriatal neurons, hippocampal neurons *in vitro* express both subtypes of dopamine receptor (Figs. 1, 2). However, it bears mentioning that cultured embryonic neurons (as used in our studies) and neostriatal cultures [19] may exhibit different patterns of dopamine receptor expression than that found in the adult nervous system [19], [32]. By employing selective D2R and D1R agonists, we showed that the inhibition of mitochondrial movement by D2R activation could be reversed by specifically activating D1 receptors, provided that the D2R agonist was washed out of the culture system. We can therefore conclude that, in hippocampal neurons, D2R activation predominates, although D1 receptors are also active.

### Akt-GSK3β is a signaling cascade downstream of dopamine in hippocampal neurons

From studies of the striatum, it is known that D1 and D2 receptors have opposing effects on adenylyl cyclase activity via coupling to the different heterotrimeric G protein subunits [24], [25]. This may explain some of the opposing effects of these two dopamine receptor subtypes that we have observed. However, it has also been recognized that the two types of dopamine receptor may have different functions due to interactions with other regulatory partners, as well as other kinds of receptors [33]–[35]. In some cases, these interactions may be independent of the cAMP pathway [11], [36].

In our studies, stimulation of each of the two receptors subtypes appeared to have opposite effects on the Akt-GSK3β signaling pathway (Fig. 7B, lanes 2 and 4), indicating that the two receptors converge on this pathway in hippocampal neurons. Given that we observed a stimulatory effect on mitochondrial movement in response to a D1R agonist, the results of our IBMX treatment experiments appear to run counter to the predicted correlation between elevated cAMP and DR1-coupled signaling, and therefore will require more thorough characterization. Nevertheless, our results suggest, first, that cAMP is probably involved in the regulation of Akt in hippocampal neurons in a manner that is different from that observed in striatal neurons (Fig. 8C) and, second, that cAMP likely plays a role in the regulation of mitochondrial movement (Fig. 9A). Our finding, that treatment with IBMX reduced levels of activated Akt in hippocampal neurons after 15 minutes, fits within the proposed temporal window of the actions of cAMP signals [11]. It is intriguing that the onset of inhibition by D2R activation occurs within the same time frame. Apropos of this fact, a plausible link between cAMP and Akt might be PP2A, a phosphatase which can be activated by PKA and is responsible for the dephosphorylation of Akt [10], [37]. Although it has been suggested that D2 receptors, and not D1 receptors, are involved in the regulation of Akt signaling pathways in the striatum [12], other studies employing cultured striatal neurons have shown that D1 receptors are also involved in the regulation of cAMP-dependent Akt signaling [26].

Axonal movement of mitochondria and other cargoes requires an association between the organelle and specific motor proteins, which, in turn, mediate movement of cargoes on cytoskeletal elements in an ATP-dependent manner [1]. Signals that alter the interactions between mitochondria and motor proteins such as KIF5, associated adapter proteins, such as miro, milton, and syntabulin, or connections between motor proteins and the cytoskeleton could have profound effects on mitochondrial movement. It has been shown that Akt can induce actin remodeling by direct phosphorylation of actin, and, notably, many cytoskeletal components are GSK3β substrates [38]. It has also been shown that Akt can be localized to mitochondria and can phosphorylate the β-subunit of ATP synthase [39], [40]. Thus, Akt may directly affect the amount of ATP locally available to drive molecular motors. Future work will be directed towards identifying the downstream targets of the Akt-GSK3β signaling cascade, which can account for the effect of dopamine on mitochondrial movement.

### The integrated effects of 5-HT and dopamine signals in the regulation of mitochondrial movement

It has been noted that 5-HT and dopamine, as well as noradrenaline, interact in a highly intricate manner [1], [41]–[44]. However, the molecular bases of these interactions have not been fully characterized. Our results reveal a novel interaction between 5-HT and dopamine that appears to involve the Akt-GSK3β signaling pathway. Dopamine and 5-HT exert opposing effects on mitochondrial trafficking in hippocampal neurons, and dopamine appears to override the stimulatory effect of 5-HT on the speed of mitochondrial movement. We hypothesize that the influence of dopamine may be important for maintaining a homeostatic distribution of mitochondria in hippocampal neurons. Consistent with this hypothesis, it has been noted in earlier studies that a homeostatic distribution of mitochondria in peripheral regions of neurons—in particular, axons and dendrites—is critical for maintaining proper function [1], [45]. Striking a balance between motile (that is, both directionally moving and oscillatory populations) and stationary populations of mitochondria may therefore be important for sustaining neuronal activity. Indeed, in the axons of hippocampal neurons, large shifts in motile and non-motile mitochondrial populations have been shown to cause dramatic changes in levels of synaptic activity [46]. Previously, we suggested that 5-HT promotes mitochondrial movement to meet localized energy requirements in regions such as the axon terminals [9]. The results of the present study suggest that dopamine may serve to limit the redistribution of potential energy sources.

A number of drugs designed to treat psychiatric disorders target the serotonergic or dopamineric systems. For example, fluoxetine, a selective serotonin reuptake inhibitor, is often prescribed for depression, and haloperidol, a D2R antagonist, has been used effectively in the treatment of schizophrenia. Together, the present study and our previous report [9] show that such drugs can affect mitochondrial movement directly via the Akt-GSK3 signaling pathway, which itself can be a direct therapeutic target, as in the administration of lithium to treat bipolar disorder. Our results therefore suggest that the integrated influence of dopamine and 5-HT signals on mitochondrial movement may be a critical factor in the maintenance of normal brain function.

## Materials and Methods

### Primary cell culture

Primary cultures of rat hippocampal neurons were prepared according to previously described methods with minor modifications [47]. Briefly, rat hippocampal cells were collected from E18 embryos and plated on poly-D-lysine (PDL)- and laminin-coated 35 mm glass-bottom dishes (MatTek, Ashland, MA) in DMEM containing high glucose (25 mM) supplemented with B27, L-asparagine, L-proline, and Vitamin B-12. This medium was used after being conditioned on monolayers of cortical glia for 24 hours. Plating densities of hippocampal neurons were 2800 cells/cm^2^ and 1200 cell/cm^2^ in conventional 35-mm dishes for protein assays and 35 mm glass-bottom dishes for imaging studies, respectively. Cells were harvested at 14 DIV for protein assays or used at 17–21 DIV in time-lapse imaging studies. On the fourth day after initial plating and every four days thereafter, Ara-C was added to control growth of glial populations. All chemical reagents were purchased from Sigma-Aldrich (St. Louis, MO) unless otherwise specified.

### Infection of cell culture

To allow visualization of mitochondria during live-cell imaging by fluorescence microscopy, cells were infected at 14 DIV with a Feline Immunodeficiency Virus (FIV)-based vector lentivirus, designated Flx1.8/CMVMitoEYFP, which encodes a MitoEYFP transgene (Clontech, Mountain View, CA). A second lentivirus was made, designated Flx1.8/CMVMitoTurboRFP, in order to label mitochondria with a red fluorescent protein (Axxora, San Diego, CA). Pseudotyped virus was generated by co-transfecting 293T cells with the transfer vector together with plasmids encoding the vesicular stomatitis virus G-glycoprotein (VSVG) and FIV gag-pol genes [48]. Viral titer was estimated by flow cytometry of infected B104 cells, and a quantity of virus was used that was sufficient to obtain an infection efficiency of 30–40%. Following infection, cells were allowed three or more days to accumulate a sufficiently strong EYFP signal before proceeding with live imaging.

### Life support for primary cultures during microscopy

To perform live-cell imaging experiments, we used a microscope stage-top incubator that we had previously designed and built to enclose a 35mm glass-bottom culture dish [9]. This incubator both provides an atmosphere of 90% air/10% CO_2_ (Airgas, Inc., San Diego, CA) and maintains ambient air and stage temperatures at 37°C. The incubator system enables us to maintain primary neuronal cultures under near-optimal conditions on the microscope stage top over many hours, or even days.

### Live cell imaging

Fluorescence microscopy was used to observe axonal transport of EYFP-labeled mitochondria in live hippocampal neurons. Time-lapse image series were acquired under high magnification (63× PLAN APO oil immersion objective; numerical aperture = 1.32; Leica, GmbH, Germany) using a Leica DMI-6000B inverted fluorescence microscope (Leica GmbH, Germany) equipped with a Sutter Lamda 10-2 emission filter wheel, Sutter DG-4 xenon light source (Sutter Instruments, Novato, CA), and a Cooke Sensiscam qe™ cooled CCD camera (Cooke Corporation, Romulus, MI). Microscope control, image capture, post-processing, and particle tracking of mitochondria were all accomplished using the Slidebook™ image acquisition and analysis software package (Intelligent Imaging Innovations, Inc., Denver, CO). In each imaging session, individual frames of mitochondria within an axon segment were acquired every 30 seconds (except where noted) for a total recording time of two hours. Cells were imaged for two hours before and two hours after administration of drugs. For masking purposes, each individual mitochondrion within a given axon segment of interest was distinguishable by its morphology (e.g., size, shape, position, and signal intensity) and behavior (e.g., pattern of movement), due in large measure to the intensity and stability of the EYFP signal.

### Image analysis

Using Slidebook™, masks of individual mitochondria were generated, and the center of area (centroid) of each mask was determined. Masks for stationary and oscillatory mitochondria were generated by Slidebook™; masks of directionally moving mitochondria were generated manually. The ID tag of each individual mitochondrion was verified by carefully reviewing movie files frame-by-frame to ensure continuity over the course of the image series, and ambiguous labels were discarded. Manual masking was performed in a double-blind procedure. The individual executing this procedure did not know whether a given time-lapse image series was acquired before treatment or after treatment. Coordinates of the centroids of masked mitochondria were then obtained using the particle tracking module within Slidebook™. The parameters used for characterizing the movement of each mitochondrion (e.g., individual label assignment, displacement, speed) were calculated from recorded changes in their X-Y coordinates. The displacement plots shown in Figs. 3–5 therefore represent the mean speeds (µm/min) of each individual mitochondrion over the full duration of each imaging session. Mean speeds are shown on the abscissa and the relative positions of individual mitochondrion along the axon segments are shown on the ordinate. Pie charts represent the initial and final proportions of each population category represented in the total pool of experiments. Speed plots represent the mean speeds of pooled directionally moving populations from all experiments, calculated at 15 minute intervals. Standard error bars represent standard errors of the mean (±SEM) calculated from the total pool of experiments; in all cases, paired Student's *t*-tests were applied to determine significance. Kymographs shown in Figure 6 were generated by the ‘smooth curve analysis’ module in Slidebook™.

### Immunohistochemical staining

EYFP-labeled hippocampal neurons cultured on glass-bottom culture dishes were fixed in 4% paraformaldehyde (pH 7.4) for 20 minutes. The cells were then permeabilized in PBT buffer (0.01% Tween-20, 0.02% BSA in PBS) for 40 minutes at room temperature. Following this, the cells were first incubated with mouse monoclonal antibody (primary antibody) for 1 hour. The cells were then incubated with a secondary fluorescein-labeled antibody for 1 hour. Finally, cells were covered in ProLong Gold® antifade reagent (Invitrogen, Carlsbad, CA) before a coverslip was applied and images were aquired. Primary antibodies used for staining were purchased from Santa Cruz Biotechnology (Santa Cruz, CA) and Chemicon (Temecula, CA). Secondary antibodies were purchased from Invitrogen (Carlsbad, CA).

### Western blotting

Western blotting was performed to determine levels of kinase activation. Cells were harvested in RIPA buffer (Tris-HCl: 50 mM, pH7.4, NP-40: 1%, Na-deoxycholate: 0.25%, NaCl: 150 mM, EDTA: 1 mM, PMSF: 1 mM, Aprotinin, leupeptin, pepstatin: 1 ug/ml each, Na_3_VO_4_: 1 mM, NaF: 1 mM). Samples were run on 10% SDS-polyacrylamide gels (Invitrogen, Carlsbad, CA). Western blotting was performed using primary antibodies to phospho-ser9-GSK3β, total GSK3β, phospho-ser473-Akt, and total Akt (Cell Signaling Technology, Danvers, MA). The secondary antibodies used for Western blotting were horseradish peroxidase (HRP)-conjugated (Jackson ImmunoResearch Laboratories, Inc., West Grove, PA), and blots were developed using ECL (GE Healthcare,Piscataway, NJ). For quantification, Western blots were scanned and intensity values were determined using Scion Image (Scion Corporation, Frederick, MD).

## Supporting Information

Figure S1 Dopamine has a sustained inhibitory effect on the activity of Akt. Western blot analysis shows that administration of dopamine (30 nM) reduced Akt activity in hippocampal neurons (as represented by the phosphorylation of serine-473 of Akt) over time.
(10.14 MB TIF)

Figure S2 D1 and D2 receptors exert opposing effects on each other. Western blot analysis shows that D1 and D2 receptor activation has opposing effects on Akt activity (as represented by the phosphorylation of serine-473) in hippocampal neurons. In these experiments, hippocampal neurons were treated with the first drug for one hour. Following washout of the first drug (receptor-specific agonist or antagonist), the second drug was applied, and after one hour, total cell lysates were collected.
(8.50 MB TIF)

Movie S1 Mitochondrial motility before treatment with Dopamine. Time-lapse series showing motility of EYFP-labeled mitochondria in cultured hippocampal neurons for two hours before administration of dopamine.
(0.77 MB MOV)

Movie S2 Mitochondrial motility after treatment with Dopamine. Time-lapse series showing motility of EYFP-labeled mitochondria in cultured hippocampal neurons for two hours after administration of dopamine.
(1.17 MB MOV)

Movie S3 Mitochondrial membrane potential after treatment with Dopamine. Time-lapse series showing stability of mitochondrial membrane potential in mitochondria labeled with the vital dye JC-1 after administration of dopamine.
(0.43 MB MOV)

Movie S4 Mitochondrial membrane potential after treatment with FCCP. Time-lapse series showing changes in mitochondrial membrane potential in mitochondria labeled with the vital dye JC-1 after administration of FCCP.
(0.29 MB MOV)

Movie S5 Mitochondrial motility before treatment with Bromocriptine, a D2R agonist. Time-lapse series showing motility of EYFP-labeled mitochondria in cultured hippocampal neurons for two hours before administration of Bromocriptine, a D2R agonist.
(1.61 MB MOV)

Movie S6 Mitochondrial motility after treatment with Bromocriptine, a D2R agonist. Time-lapse series showing motility of EYFP-labeled mitochondria in cultured hippocampal neurons for two hours after administration of Bromocriptine, a D2R agonist.
(1.01 MB MOV)

Movie S7 Mitochondrial motility before treatment with Haloperidol, a D2R antagonist. Time-lapse series showing motility of EYFP-labeled mitochondria in cultured hippocampal neurons for two hours before administration of Haloperidol, a D2R antagonist.
(5.45 MB MOV)

Movie S8 Mitochondrial motility after treatment with Haloperidol, a D2R antagonist. Time-lapse series showing motility of EYFP-labeled mitochondria in cultured hippocampal neurons for two hours after administration of Haloperidol, a D2R antagonist.
(9.48 MB MOV)

Movie S9 Mitochondrial motility before treatment with SKF38393, a D1R agonist. Time-lapse series showing motility of EYFP-labeled mitochondria in cultured hippocampal neurons for two hours before administration of SKF38393, a D1R agonist.
(3.58 MB MOV)

Movie S10 Mitochondrial motility after treatment with SKF38393, a D1R agonist. Time-lapse series showing motility of EYFP-labeled mitochondria in cultured hippocampal neurons for two hours after administration of SKF38393, a D1R agonist.
(3.73 MB MOV)

Movie S11 Mitochondrial motility before treatment with SCH23390, a D1R antagonist. Time-lapse series showing motility of EYFP-labeled mitochondria in cultured hippocampal neurons for two hours before administration of SCH23390, a D1R antagonist.
(6.55 MB MOV)

Movie S12 Mitochondrial motility after treatment with SCH23390, a D1R antagonist. Time-lapse series showing motility of EYFP-labeled mitochondria in cultured hippocampal neurons for two hours after administration of SCH23390, a D1R antagonist.
(1.22 MB MOV)

Movie S13 Mitochondrial motility before treatment with Bromocriptine. Time-lapse series showing basal motility of EYFP-labeled mitochondria in cultured hippocampal neurons for two hours before administration of Bromocriptine.
(3.15 MB MOV)

Movie S15 Mitochondrial motility following washout - first hour. Time-lapse series showing mitochondrial motility in the first hour following the washout of bromocriptine and subsequent treatment with SKF38393, a D1 agonist.
(0.24 MB MOV)

Movie S14 Mitochondrial motility after treatment with Bromocriptine. Time-lapse series showing mitochondrial motility following treatment with bromocriptine.
(0.86 MB MOV)

Movie S16 Mitochondrial motility following washout - second hour. Time-lapse series showing mitochondrial motility in the second hour following washout of Bromocriptine and treatment with SKF38393.
(0.52 MB MOV)

Movie S17 Mitochondrial motility before treatment with IBMX. Time-lapse series showing mitochondrial motility before treatment with IBMX.
(3.71 MB MOV)

Movie S18 Mitochondrial motility after treatment with IBMX. Time-lapse series showing mitochondrial motility following treatment with IBMX.
(0.71 MB MOV)
