# Correction: Dopamine Inhibits Mitochondrial Motility in Hippocampal Neurons

**DOI:** 10.1371/annotation/dcde3f9c-4be2-40a0-b9a2-152f6772fb6d

**Published:** 2008-08-11

**Authors:** Sigeng Chen, Geoffrey C. Owens, David B. Edelman

Figures 8 and 9 appeared out of order. Please view the correct Figure 8 with its legend here:

**Figure 8:**
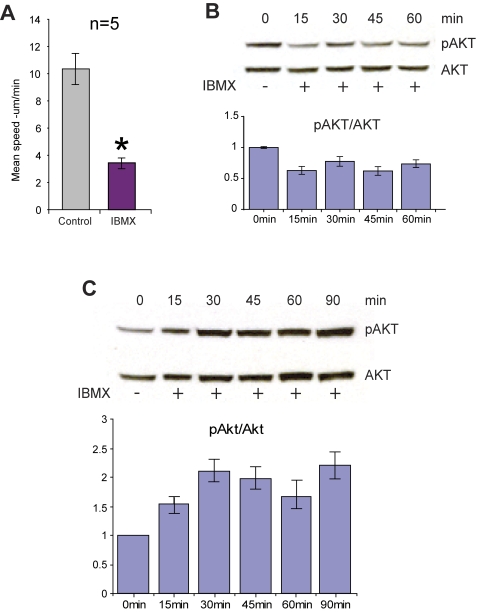
Treatment with IBMX has different effects on hippocampal neurons and striatal neurons (A-C). A. Inhibition of mitochondrial movement by IBMX. Mean speed (μm/min) of all directionally moving mitochondria from pooled experiments before and after treatment with IBMX (100 μM; n = 5, paired *t*-test; p<0.02). B. Western blot analysis shows that administration of IBMX (100 μM) reduces Akt activity in hippocampal neurons, which is represented by decreased levels of phosphorylated serine-473 of Akt, relative to total Akt, over time. C. Western blot analysis shows that administration of IBMX (100 μM) increases Akt activity in striatal neurons, which is represented by decreased levels of phosphorylated serine-473 of Akt, relative to total Akt, over time.

