# Correction: Dopamine Inhibits Mitochondrial Motility in Hippocampal Neurons

**DOI:** 10.1371/annotation/07edbe56-d503-477f-adcf-d7ec30e9beda

**Published:** 2008-08-11

**Authors:** Sigeng Chen, Geoffrey C. Owens, David B. Edelman

Figures 8 and 9 appeared out of order. Please view the correct Figure 9 with its legend here:

**Figure 9:**
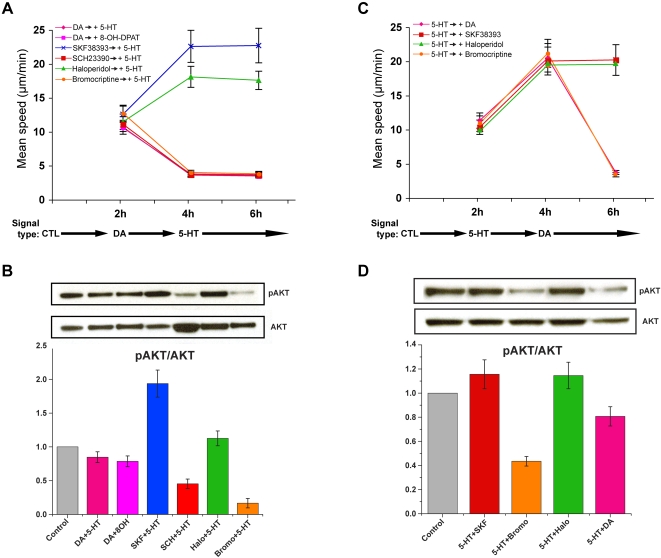
Dopamine and 5-HT exert opposing effects on mitochondrial movement and Akt activity (A-D). Mean speeds of directionally moving mitochondrial populations are shown at two hour intervals in A and C. Akt activity levels in cultures after treatment with DA and 5-HT are shown in B and D. Mitochondrial movement was measured following serial administration of dopamine or its receptor agonists, antagonists, and 5-HT. In these experiments, a 6 hour period of observation was divided into three two-hour intervals, each allocated for a different treatment, i.e., control (CTL), dopamine (DA), or 5-HT. A. Prior to treatment with 5-HT, hippocampal neurons were pretreated with dopamine receptor-specific agonists or antagonists, including DA, SKF38393, SCH2390, haloperidol, and bromocriptine. B. Western blot analysis shows that dopamine counteracts the effect of 5-HT on Akt activity in the presence of 5-HT. C. Prior to treatment with dopamine signals, hippocampal neurons were pretreated with 5-HT. Each mean speed value in the graph represents three repeat experiments, i.e., separately prepared cultures (n = 3, paired *t*-test; p<0.02; 103–130 mitochondria were tracked in calculating the average speeds of each set of mitochondria in a given treatment interval). D. Western blot analysis shows that 5-HT failed to counteract the effect of dopamine on Akt activity in the presence of dopamine. All other conditions are the same as previously described in Figs. 3-5.

