# Supplementary figures and images for: Dopamine Inhibits Mitochondrial Motility in Hippocampal Neurons
Source: PLoS One. 2008 Jul 30;3(7):e2804. doi: 10.1371/journal.pone.0002804 (PMC2467486; doi:10.1371/journal.pone.0002804)

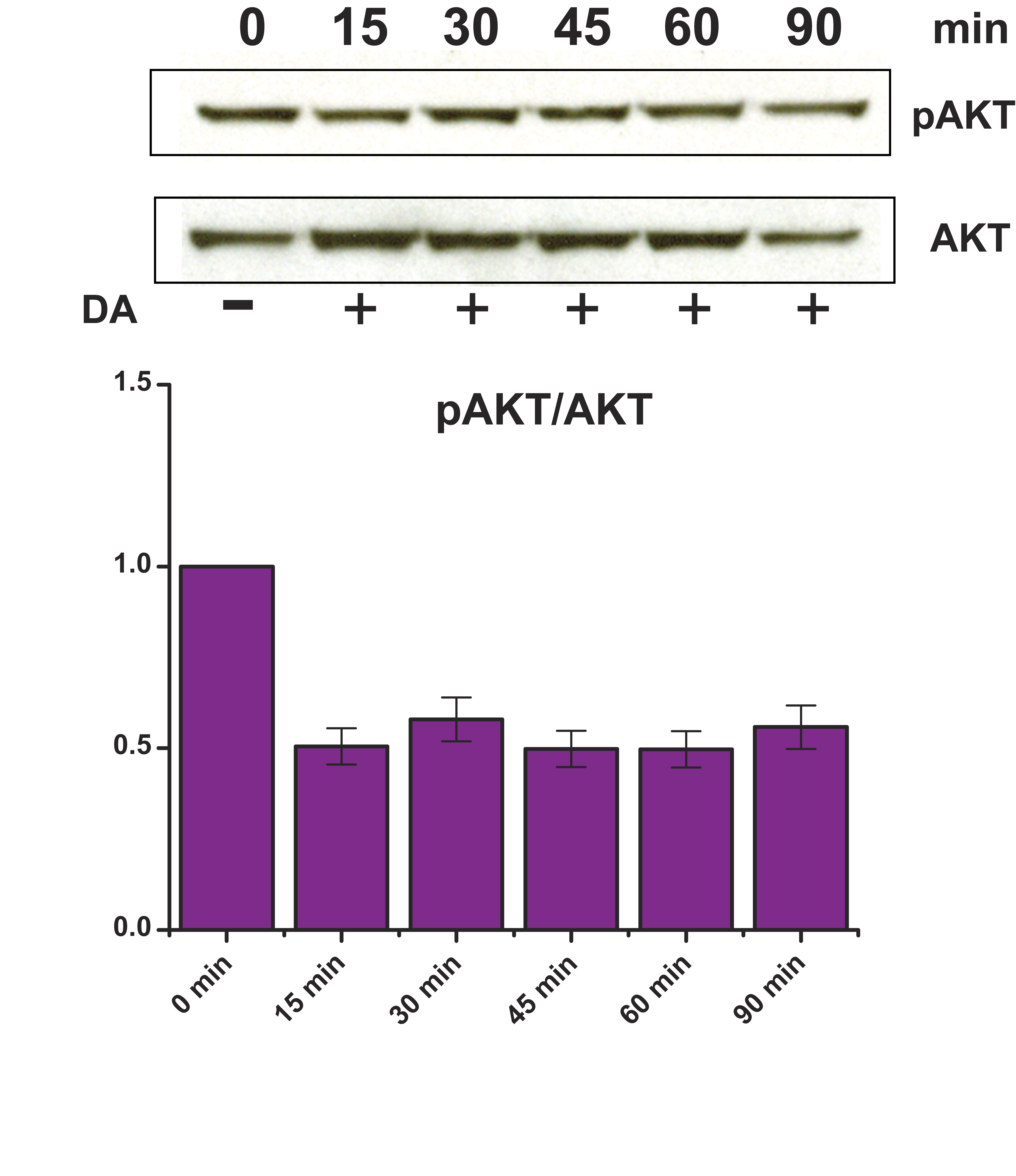

Supplement: Figure S1 — Dopamine has a sustained inhibitory effect on the activity of Akt. Western blot analysis shows that administration of dopamine (30 nM) reduced Akt activity in hippocampal neurons (as represented by the phosphorylation of serine-473 of Akt) over time. (10.14 MB TIF) [file pone.0002804.s001.tif]

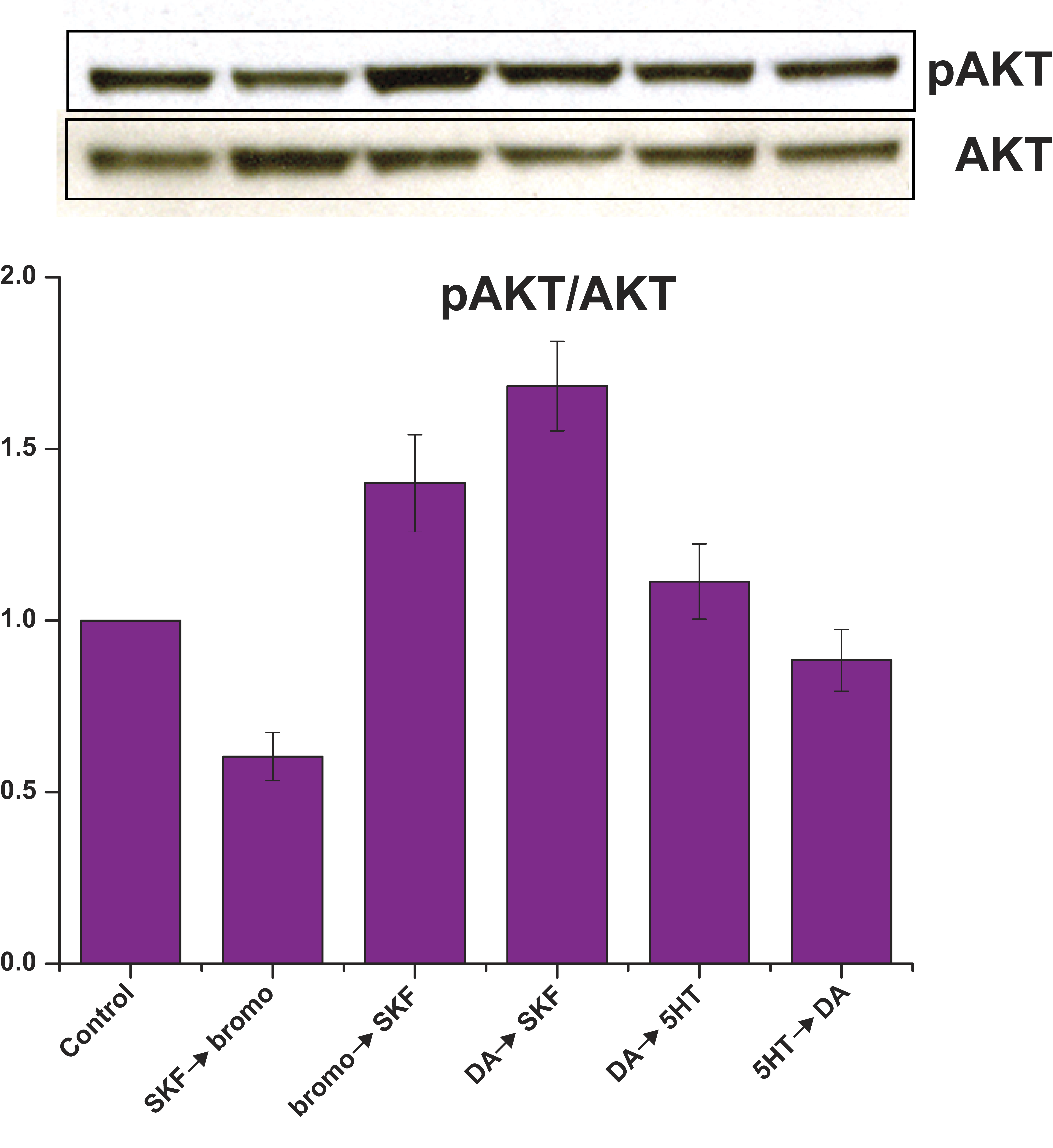

Supplement: Figure S2 — D1 and D2 receptors exert opposing effects on each other. Western blot analysis shows that D1 and D2 receptor activation has opposing effects on Akt activity (as represented by the phosphorylation of serine-473) in hippocampal neurons. In these experiments, hippocampal neurons were treated with the first drug for one hour. Following washout of the first drug (receptor-specific agonist or antagonist), the second drug was applied, and after one hour, total cell lysates were collected. (8.50 MB TIF) [file pone.0002804.s002.tif]
